# Evidence and impact of map error on land use and land cover dynamics in Ashi River watershed using intensity analysis

**DOI:** 10.1371/journal.pone.0229298

**Published:** 2020-02-20

**Authors:** Vitus Tankpa, Li Wang, Raphael Ane Atanga, Alfred Awotwi, Xiaomeng Guo

**Affiliations:** 1 State Key Laboratory of Urban Water Resource and Environment, School of Environment, Harbin Institute of Technology, Harbin, China; 2 Department of Tourism, School of Tourism and Hospitality, University of Johannesburg, Johannesburg, South Africa; 3 Department of Environmental Science, University for Development Studies (UDS), Navrongo, Ghana; National Institute of Geophysics, Geodesy and Geography, BULGARIA

## Abstract

Previously, applications of intensity analysis (IA) on land use and land cover change (LULCC) studies have focused on deviations from uniform intensity (UI) and failed to quantify the reasons behind these deviations. This study presents the application of IA with hypothetical errors that could explain non-uniform LULCC in the context of IA at four-time points. LULCC in the Ashi watershed was examined using Landsat images from 1990, 2000, 2010 and 2014 showing the classes: Urban, water, agriculture, close canopy, open canopy and other vegetation. Matrices were created to statistically examine LULCC using IA. The results reveal that the seeming LULCC intensities are not uniform with respect to the interval, category and transition levels of IA. Error analysis indicates that, hypothetical errors in 13%, 19% and 11.2% of the 2000, 2010 and 2014 maps respectively could account for all differences between the observed gain intensities and the UI; while errors in 12%, 21%, and 11% of the 1990, 2000 and 2010 maps respectively could account for all differences between the observed loss intensities and the UI. A hypothetical error in 0.6% and 1.6% of the 1990 map; 1.5% and 4% of the 2000 map; 1.2% and 2.1% of the 2010 map could explain divergences from uniform transitions given URB gain and AGR gain during 1990–2000, 2000–2010 and 2010–2014 respectively. Evidence for a specific deviation from the relevant hypothesized UI is either strong or weak depending on the size of these errors. We recommend that users of IA concept consider assessing their map errors, since limited ground information on past time point data exist. These errors will indicate strength of evidence for deviations and reveals patterns that increase researcher’s insight on LULCC processes.

## 1. Introduction

The examination of land use and land cover change (LULCC) at a watershed scale is pivotal due to the function land use and land cover (LULC) provides. This examination is particularly a concern due to the impact of urbanization and agricultural activities in watersheds [1]. Globally, urban and agricultural systems are challenged by their impact on the local environment [2, 3]. Changes to watershed landscapes by urban sprawl, agriculture and loss of forest are increasingly of interest [4, 5]. These developments in a watershed result in LULCC patterns which affect hydrologic processes and transfer non-point source pollutants [6–8]. LULC is associated with sustainable management of water resources as it influences hydrological processes and water quality [9, 10]. There is therefore, a need for research to examine the forms and manner in which LULC changes because of urbanization and agriculture in developing countries, where urban areas are projected to grow from 300,000km^2^ to 1,200,000 km^2^ between 2000 and 2050 [11]. Accurately assessing map errors to reveal patterns that complement analysis of systematic process on how LULCC might evolve at the watershed scale is empirically valuable for water resources assessment and land-based pollution management [12, 13].

After the launch of Landsat-1 in 1972 and following the arrival of geospatial techniques, studies in LULCC depend on a cross tabulation matrix to investigate LULCC. The basis of various researches in LULCC depends on these matrices [14–19]. Comparison among the elements in the matrix of these studies fail to reveal the observed patterns that could result from processes that are methodically more intensive or less intensive than seemingly random or uniform processes [20]. Nevertheless, the use of these matrices helps scientists recognize some significant patterns of changes.

The importance of information on the systematic transition of LULCC for strategic land-based pollution management, necessitated scientists to devise a unified method that quantifies systematic processes of LULCC as proposed [21]. The traditional matrix methods was further developed into a unified approach named intensity analysis (IA) [20], which examines changes among LULC types on three levels; time interval, category and transition. This approach allows researchers quantify the deviations or differences between each detected alteration intensity and a hypothesized uniform alteration intensity for each analysis. Based on this, [22] improved on their methods to further quantify errors that could account for the differences for each level of the intensity analysis (IA). A clear description on these errors have been presented [23, 24].

Several authors [3, 25–33] across Africa, Asia, Australia, Europe, North America and South America have used the concept of IA to gain insight into processes of LULCC without considering errors that could explain the deviations. Besides, limited applications measured how various levels of errors in their data could influence the results and interpretation at each level of the IA, since scientists do not have control over accurate errors in their maps. This study fills this gap by applying and testing the improved version of IA framework [22] at the watershed scale. Additionally, the research captures deviations at each level of the analysis and finds out the practical implication of this method.

After China’s 1978 market-oriented reforms, the study site was exposed to intensive agriculture, urbanization and deforestation, which led to pollution of the Ashi river, the main river of the watershed [34, 35]. Nonetheless, studies that can offer insight into LULCC processes using the IA concept are not yet reported. Producing science-based evidence from such studies can help land and water resource managers to recognize patterns of LULCC and assess the extent of stationarity of the changes over time. This study maps out, analyzes the LULCC of the Ashi watershed from 1990 to 2014, and applies the modified version of the IA method. It seeks to answer the following questions: Is the yearly LULC changes in the watershed uniform across time intervals; are the gains and losses of LULC types uniform throughout the watershed; and is the transition of the losing classes even across the watershed, given a specific class gain. The main objective is to detect the LULCC intensity from 1990 to 2014 under three intervals being 1990–2000, 2000–2010 and 2010–2014, and to measure how various levels of map errors in the data could influence the intensity of change of the watershed.

## 2. Materials and methods

### 2.1 Materials

#### 2.1.1 Study area

**Fig 1:**
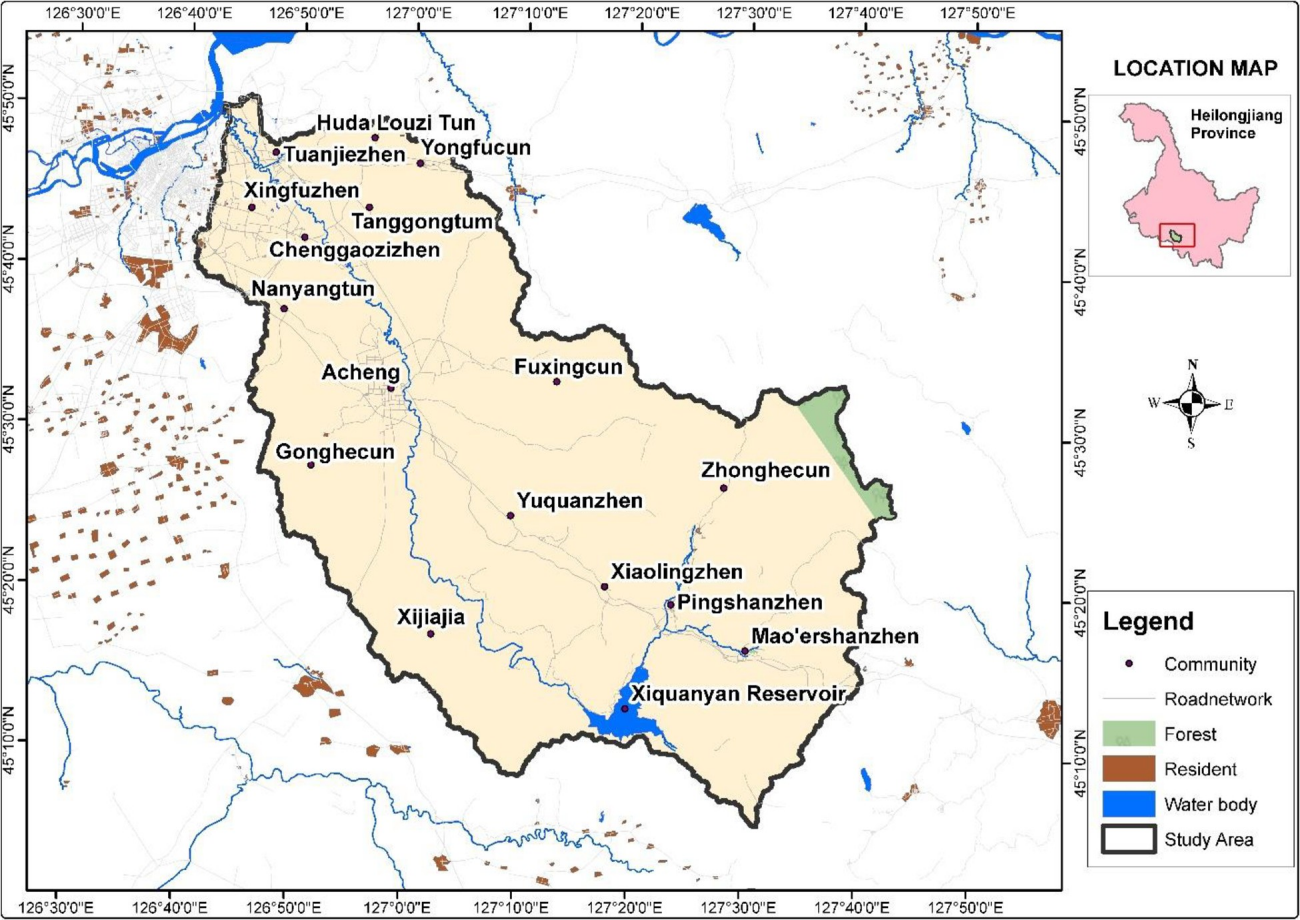
Ashi River Watershed located in the south-west of Heilongjiang Province.

The coverage area of the Ashi watershed is 3,545 km^2^, in the southwest of Heilongjiang Province, northeast China. It is confined by latitude 45°05'–45°49'N, and longitude 126°40'–127°42’E (Fig 1). The focal waterway of the watershed which functions as a tributary of the Songhua River is the Ashi River, spanning 213 km.The watershed altitude above sea level is between 109 to 833 m, with slopes ranging from 0 to 67.3%. Northwest of the watershed is flat, while the southeast has low-lying hilly and sloping vegetation. The watershed experiences nippy atmosphere in the winter, with a mean temperature of 3.4°C and minimum temperature of– 40°C. The watershed witnesses winter between November and mid-April. The zone gets uneven precipitation, which peaks in July and August. The multi-year normal precipitation is 580–600 mm [34, 36].

#### 2.1.2 Data

Four Landsat satellite images (1990, 2000, 2010 and 2014) were acquired to study the trend of LULCC between 1990 and 2014. Fieldwork was implemented during the dry season with Global Position System (GPS) to take coordinates for each LULC category and aid classification. This was supplemented by points collected over historical images archived in Google Earth. 30 m Landsat satellite surface reflectance images were acquired in zipped files from the US Geological Survey (USGS) Earth Explorer site (http://glovis.usgs.gov) and extracted to assess temporal and spatial changes in the watershed. The Ashi watershed falls in one Landsat path (117) and two rows (28 and 29). The images were captured with Landsat 7 thematic mapper (TM)/Enhanced thematic mapper plus (ETM+) and Landsat 8 operational land imager (OLI).

### 2.2 Methods

#### 2.2.1 Image processing and classification

LULC was mapped in four (4) different periods; 1990, 2000, 2010 and 2014 using spectral variations exhibited by the features in the watershed. Top-of-atmosphere (TOA) calibration was used to convert the Digital Number (DN) values to reflectance in order to minimize the radiometric differences caused by disparities in TM and Landsat 8 sensors because of sensor-target-illumination. The images were geometrically corrected and changed to TOA reflectance by means of information provided in the metadata [37]. Two scenes covered the watershed, and for each period, atmospherically calibrated images were mosaicked and clipped to the extent of the watershed boundary under study. Supervised classification with maximum likelihood algorithm was used to classify the images. Six LULC classes were discovered namely URB, AGR, WAT, CLC, OPC and OTV (Table 1).

**Table 1:** Classes defined based on supervised classification.

Class	Description
Urban (URB)	Impervious surfaces; residential, industrial, transportation, communications, commercial and bare surfaces
Water (WAT)	Rivers, Lakes and Ponds
Agriculture (AGR)	Pasture, cropland (rice), orchards and fallow land
Closed Forest (CLC)	Areas with mature trees growing close together
Open Forest (OPC)	Areas with a partial disturbance in tree canopy either through logging or burning
Other vegetation (OTV)	Includes all vegetation features that are not typical of forest and agriculture. e.g. grassland/shrubs with very few scattered trees

#### 2.2.2 Post-classification refinement

Using ancillary DEM data, the classified images were improved by setting elevation thresholds to separate water areas from shadows in forest areas. Normalized differencing between NIR/Red and Green/NIR were used to generate Normalized Difference Vegetation Index (NDVI) and Normalized Difference Water Index (NDWI). Thresholds were set to highlight water, built and vegetation cover. Coupling these with DEM, the results from the supervised classifications were refined. NDVI=NIR−RedNIR+Red, $NDWI=\frac{Green-NIR}{Green+NIR}$

Finally, a 3x3 spatial filter was used on each thematic image to reduce “salt and pepper” effect caused by isolated pixels. The filter was used to notice isolated pixels and assign them to the dominant class in their location. Area statistics for each information class was calculated in hectares by multiplying the area per pixel and number of pixels in a class.

#### 2.2.3 Intensity analysis (IA)

Intensity analysis is a quantitative framework that examines LULCC of an area from one time point to another. It summarizes changes for each time interval and involves the intensity of LULC conversion processes at time intervals, category and transition levels. Detected rate of changes is compared with a uniform rate of changes that would occur if yearly speed of the changes were distributed uniformly throughout the whole temporal and spatial extend. This concept unifies all time phases for a complete LULCC assessment [20]. IA is used based on the transition matrix created from the study site to perform deep analysis of the LULCC of the watershed. This study uses 8 equations with notation shown in Table 2 [20].

**Table 2:** Mathematical notation following Aldwalk and Pontius (2012, 2013).

*Symbol*	*Description*
*T*	Number of time points
*Y_t_*	year at time point t
*t*	index for the initial time point of interval [Y_t,_ Y_t +1_], where t ranges from 1 to T-1
*J*	number of classes, which is equal to 6 in our case study
*i*	index for a class at an interval's initial time point
*j*	index for a class at an interval's final time point
*m*	index for the losing class for selected transition
*n*	index for gaining class for the selected transition
*C_tij_*	number of elements that transition from class i to class j during interval [Y_t,_ Y_t+1_]
*S_t_*	annual change during interval [Y_t_, Y_t+1_]
*U*	uniform annual change during extent[Y_1,_Y_T_]
*G_tj_*	intensity of annual gain of class j during interval [Y_t,_ Y _t+1_] relative to size of class j at time t+1
*L_ti_*	intensity of annual loss of class i during interval [Y_t,_ Y _t+1_] relative to size of class i at time t
*R_tin_*	intensity of annual transition from class i to class n during interval [Y_t,_ Y_t+1_] relative to size of class i at time t
*W_tn_*	uniform intensity of annual transition from all non-n classes to class n during interval [Y_t,_Y_t+1_] relative to size of all non-n classes at time t
*Q_tmj_*	intensity of annual transition from class m to class j during interval [Y_t,_ Y_t+1_] relative to size of class j at time t+1
*V_tm_*	uniform intensity of annual transition from class m to all non-m classes n during interval [Y_t,_Y_t+1_] relative to size of all non-m classes at time t +1
*E^s^_t_*	commission of change error during interval [Y_t,_ Y_t+1_], i.e., percent of domain that is observed change during interval [Y_t,_ Y_t+1_] but is hypothesized persistence
*O_t_^s^*	omission of change error during interval [Y_t,_ Y_t+1_], i.e., percent of domain that is observed persistence during interval [Y_t,_ Y_t+1_] but is hypothesized change
*E^G^_tj_*	commission of class j error at time t+1, i.e., number of elements that are observed gains of category j during interval [Y_t_, Y_t+1]_ but are hypothesized gains of a non-j class
*O^G^_tj_*	omission of class j error at time t+1,i.e., number of elements that are observed gains of a non-j class during interval [Y_t,_ Y_t+1_] but are hypothesized gains of class j
*E^L^_ti_*	commission of class i error at time t, i.e., number of elements that are observed losses of class i during interval [Y_t,_ Y_t+1_] but are hypothesized losses of a non-i class
*O^L^_ti_*	omission of class i error at time t,i.e., number of elements that are observed losses of a non-i class during interval [Y_t,_ Y_t+1_] but are hypothesized losses of class i
*E^R^_tin_*	commission of class i error at time t, i.e., number of elements that are observed transitions from class i to class n during interval [Y_t,_ Y_t+1_] but are hypothesized transitions from a non-i class to class n
*O^R^_tin_*	omission of class i error at time t,i.e., number of elements that are observed transition from a non-i class to class n during interval [Y_t_, Y_t+1_] but are hypothesized transitions from class i to class n
*E^Q^_tmj_*	commission of class j error at time t+1, i.e., number of elements that are observed transtions from class m to category j during interval [Y_t,_ Y_t+1_] but are hypothesized transitions from class m to a non-j class
*O^Q^_tmj_*	omission of class j error at time t+1,i.e., number of elements that are observed transitions from class m to a non-j class during interval [Y_t,_ Y_t+1_] but are hypothesized transitions from class m to class j

The time interval level compares the size and yearly rate of change S_t_ during each time interval [Y_t_, Y_t+1_] to a uniform yearly rate of change U during the time extent [Y_1_,Y_T_]. At this stage, IA assumes that U is equal to the actual yearly changes during all time intervals. If S_t_ < U, then it implies the interval [Y_t_, Y_t+1_] had slow change. If S_t_ > U, then it implies the interval [Y_t_, Y_t+1_] had fast change as defined by IA. Eqs (1) and (2) calculate S_t_ and U respectively and assumes that the area extent is alike at each time point.

$$S_{t}=\frac{\sum_{j=1}^{J}\left[\left(\sum_{i=1}^{J}C_{tij}\right)-C_{tjj}\right]}{\left(Y_{t+1}-Y_{t}\right)\left(\sum_{j=1}^{J}\sum_{i=1}^{J}C_{tij}\right)}100\%$$ (1)

$$U=\frac{\sum_{t=1}^{T-1}\left\{\left(Y_{t+1}-Y_{t}\right)\sum_{j=1}^{J}\left[\left(\sum_{i=1}^{J}C_{tij}\right)-C_{tjj}\right]\right\}}{\left(Y_{T}-Y_{1}\right)\sum_{t=1}^{T-1}\left[\left(Y_{t+1}-Y_{t}\right)\left(\sum_{j=1}^{J}\sum_{i=1}^{J}C_{tij}\right)\right]}100\%$$ (2)

The category level investigates whether the gain or loss of a class is dormant or active during each time interval. It calculates the intensity of yearly gross gains and losses using Eqs (3) and (4) respectively for each class in each time interval and compares them with a uniform intensity, S_t,_ of gains and loss for all classes. If the value of Eq (3) > Eq (1), then the gain of class j is active but if the value of Eq (3) < Eq (1), then the gain of class j is dormant. The same approach to gains also applies to losses. Eq (1) associates the interval level investigation with the category level investigation for each time interval.

$$G_{tj}=\frac{\left[\left(\sum_{i=1}^{J}C_{tij}\right)-C_{tjj}\right]/\left(Y_{t+1}-Y_{t}\right)}{\sum_{i=1}^{J}C_{tij}}100\%$$ (3)

$$L_{ti}=\frac{\left[\left(\sum_{j=1}^{J}C_{tij}\right)-C_{tii}\right]/\left(Y_{t+1}-Y_{t}\right)}{\sum_{j=1}^{J}C_{tij}}100\%$$ (4)

The transition level compares the intensity of yearly transition R_tin_ from class i to class n to a hypothesized or uniform intensity of yearly transition, W_tn,_ given the gain of class n during interval [Y_t_,Y_t+1_]. Eqs (5) and (6) calculate R_tin_ and W_tn_ respectively. If the value of Eq (5) < Eq (6), then gain of n avoids i, implying that the area gained by n from i is small during [Y_t_,Y_t+1_] than if the area gained by n were to have transitioned from the area that is not n at time Y_t_ uniformly. If the value of Eq (5) > Eq (6) the gain of n targets i, implying the area gained by n from i is more intensive during [Y_t_,Y_t+1_] than if the gain of n were to have transitioned from the area that is not n at time Y_t_ uniformly. It also examines the loss of class i to n due to the transition from i to n; Eqs (7) and (8) investigate the loss of class i in the same manner that Eqs (5) and (6) investigates the gain of n. This study investigated the transition from CLC, OPC, OTV and WAT to URB and AGR and also the losses of these LULC classes due to their transition to URB and AGR.

$$R_{tin}=\frac{C_{tin}/\left(Y_{t+1}-Y_{t}\right)}{\sum_{j=1}^{J}C_{tij}}100\%$$ (5)

$$W_{tn}=\frac{\left[\left(\sum_{i=1}^{J}C_{tin}\right)-C_{tnn}\right]/\left(Y_{t+1}-Y_{t}\right)}{\sum_{j=1}^{J}\left[\left(\sum_{i=1}^{J}C_{tij}\right)-C_{tnj}\right]}100\%$$ (6)

$$Q_{tmj}=\frac{C_{tmj}/\left(Y_{t+1}-Y_{t}\right)}{\sum_{i=1}^{J}C_{tij}}100\%$$ (7)

$$V_{tm}=\frac{\left[\left(\sum_{j=1}^{J}C_{tmj}\right)-C_{tmm}\right]/\left(Y_{t+1}-Y_{t}\right)}{\sum_{i=1}^{J}\left[\left(\sum_{j=1}^{J}C_{tij}\right)-C_{tim}\right]}100\%$$ (8)

#### 2.2.4 Error analysis

To find out whether map errors could explain the divergence from the hypothesized uniform intensities revealed by IA, assuming the hypothesized uniform intensities were true, the study used the modified version of [20] to calculate the least hypothetical error that could account for the difference between hypothesized UI and change intensity of each level of the IA [22]. These errors are computed based on a null hypothesis that the change intensities are uniform, where map error could account for each difference between detected change intensity and uniform intensity [22, 23]. Weak evidence is given by small hypothetical error while strong evidence is given by large error against this null hypothesis. At the interval level, Eqs (9–14) calculate the error in the map that can explain the difference between S_t_ and U. Eqs (9, 10 and 11) apply where S_t_ > U and Eqs (12, 13 and 14) apply where S_t_ < U.

$$E_{t}^{S}=\left(S_{t}-U\right)\left(Y_{t+1}-Y_{t}\right)$$ (9)

Observed change during interval

$$t=U\;\left(Y_{t+1}-Y_{t}\right)+E_{t}^{S}$$ (10)

Commission of change intensity during

$$\left[Y_{t},Y_{t+1}\right]=\frac{E_{t}^{S}}{S_{t}\left(Y_{t+1}-Y_{t}\right)}100\%$$ (11)

$$O_{t}^{S}=\left(U-S_{t}\right)\left(Y_{t+1}-Y_{t}\right)$$ (12)

Uniform change during interval

$$t=S_{t}\left(Y_{t+1}-Y_{t}\right)+O_{t}^{S}$$ (13)

Omission of change intensity during

$$\left[Y_{t},Y_{t+1}\right]=\frac{O_{t}^{S}}{S_{t}\left(Y_{t+1}-Y_{t}\right)+O_{t}^{S}}100\%$$ (14)

The error that could explain for the difference (G_tj_-S_t_) in the map of the final period is computed by Eqs (15, 16, 17 and 18) at the category stage. Classes where G_tj_ > S_t_ are computed by Eqs (15 and 16). Eq (15) computes the area gained by class j that is assumed to be an error of commission of class j at the final period. A study [17] in IA produces the source of Eqs (15, 17, 19, 20, 26, 27, 28 and 30) that follow the same reasoning. Eq (16) calculates the intensity of commission of class j error at the final period, where class j error is committed by the numerator in Eq (16) and the denominator is the size of area gained by class j. Classes where G_tj_ < S_t_ are calculated by Eqs (17 and 18). Eq (17) calculates the area gained by class j with the assumption that it is omission of class j error at the final period and the intensity of error omitted is calculated by Eq (18). Eqs (19, 21, 20 and 22) computes the error that could explain the difference (L_t_ -S_t_). If L_ti_ > S_t_, Eq (19) computes the area loss by class i that is assumed to be an error committed by class i at the early period. Eq (21) calculates the intensity of commission of class i error at the early period, where the class i mistake is committed by the numerator in Eq (21) and the denominator is the size of area loss by class i. Eqs (20 and 22) apply to classes where L_ti_ < S_t_. Eq (20) computes area loss by class i that is assumed to be omission of class i error at the early period. Eq (22) expresses the intensity of omission of class i error at the early period. Eq (23) gives the minimum assumed error at the initial time point expressed as a proportion of the interval’s watershed area that could account for all deviations between S_t_ and G_tj_ for all classes.

$$E_{tj}^{G}=\frac{\left(\sum_{i=1}^{J}C_{tij}\right)\left(Y_{t+1}-Y_{t}\right)\left(G_{tj}-S_{t}\right)}{100\%-\left(Y_{t+1}-Y_{t}\right)S_{t}}$$ (15)

Commission of j intensity at

$$t+1=\frac{E_{tj}^{G}}{\left(\sum_{i=1}^{J}C_{tij}\right)-C_{tjj}}100\%$$ (16)

$$O_{tj}^{G}=\frac{\left(\sum_{i=1}^{J}C_{tij}\right)\left(Y_{t+1}-Y_{t}\right)\left(S_{t}-G_{tj}\right)}{100\%-\left(Y_{t+1}-Y_{t}\right)S_{t}}$$ (17)

Omission of j intensity at

$$t+1=\frac{O_{tj}^{G}}{\left[\left(\sum_{i=1}^{J}C_{tij}\right)-C_{tjj}\right]+O_{tj}^{G}}100\%$$ (18)

$$E_{ti}^{L}=\frac{\left(\sum_{j=1}^{J}C_{tij}\right)\left(Y_{t+1}-Y_{t}\right)\left(L_{ti}-S_{t}\right)}{100\%-\left(Y_{t+1}-Y_{t}\right)S_{t}}$$ (19)

$$O_{ti}^{L}=\frac{\left(\sum_{j=1}^{J}C_{ti}\right)\left(Y_{t+1}-Y_{t}\right)\left(S_{t}-L_{ti}\right)}{100\%-\left(Y_{t+1}-Y_{t}\right)S_{t}}$$ (20)

Commission of i intensity at

$$t=\frac{E_{ti}^{L}}{\left(\sum_{j=1}^{J}C_{tij}\right)-C_{tii}}100\%$$ (21)

Omission of i intensity at

$$t=\frac{O_{ti}^{L}}{\left[\left(\sum_{j=1}^{J}C_{tij}\right)-C_{tii}\right]+O_{ti}^{L}}100\%$$ (22)

Error at *t* as % of watershed area

$$=\frac{\sum_{i=1}^{J}E_{ti}^{L}}{\sum_{j=1}^{J}\sum_{i=1}^{J}C_{tij}}\times 100\%=\frac{\sum_{i=1}^{J}O_{ti}^{L}}{\sum_{j=1}^{J}\sum_{i=1}^{J}C_{tij}}100\%$$ (23)

Minimum error that could account for all departures in intensities for all non-n classes from the uniform transition intensity is calculated on assumption of commission error of a targeted class and omission error of an avoided class for each error. Precisely, if a gain by class n seems to target class i, then commission of class i error is hypothesized at the early period in the area that has omission of an avoided class. The area assumed to have error of commission of class i at the early time is calculated by Eq (26), which would explain the area gained by n targeting i, that is where R_tin_>W_tn._ Eq (26) becomes zero if the area gained by n does not target i. Eq (24) computes the commission of intensity. Likewise, if the gain of class n seems to avoid class i, then omission of class i error is hypothesized at the early period in the area that has commission of a targeted class. The area assumed to have error of omission of class i at the early time is calculated by Eq (27), which could explain the area gained by n avoiding i, where R_tin_<W_tn._ Eq (27) becomes zero if area gained by n does not avoid i. The intensity of this error is estimated by Eq (25). In a similar fashion, IA compares Q_tmj_ to V_tm._ If Q_tmj_ > V_tm_ then class m loss targets class j. If Q_tmj_ < V_tm_ then class m loss avoids class j. Eq (28) gives the commission of class j error at the final period point that could account for the deviation (Qtmj—V_tm_) where Q_tmj_ > V_tm_. The intensity of the error is given by Eq (29). If Q_tmj_ < V_tm_ Eq (30) calculates the omission of class j error at the final period that could account for the deviation. Eq (31) gives the intensity of that error.

Commission of i intensity at

$$t=\frac{E_{tin}^{R}}{C_{tin}}100\%$$ (24)

Omission of i intensity at

$$t=\frac{O_{tin}^{R}}{O_{tin}^{R}+C_{tin}}100\%$$ (25)

$$E_{tin}^{R}=\frac{\left(\sum_{j=1}^{J}C_{tij}\right)\left(Y_{t+1}-Y_{t}\right)\left(R_{tin}-W_{tin}\right)}{100\%-\left(Y_{t+1}-Y_{t}\right)W_{tn}}$$ (26)

$$O_{tin}^{R}=\frac{\left(\sum_{j=1}^{J}C_{tij}\right)\left(Y_{t+1}-Y_{t}\right)\left(W_{tn}-R_{tin}\right)}{100\%-\left(Y_{t+1}-Y_{t}\right)W_{tn}}$$ (27)

$$E_{tmj}^{Q}=\frac{\left(\sum_{i=1}^{J}C_{tij}\right)\left(Y_{t+1}-Y_{t}\right)\left(Q_{tmj}-V_{tm}\right)}{100\%-\left(Y_{t+1}-Y_{t}\right)V_{tm}}$$ (28)

Commission of j intensity at

$$t+1=\frac{E_{tmj}^{Q}}{C_{tmj}}100\%$$ (29)

$$O_{tmj}^{Q}=\frac{\left(\sum_{i=1}^{J}C_{tij}\right)\left(Y_{t+1}-Y_{t}\right)\left(V_{tm}-Q_{tmj}\right)}{100\%-\left(Y_{t+1}-Y_{t}\right)V_{tm}}$$ (30)

Omission of j intensity at

$$t+1=\frac{O_{tmj}^{Q}}{O_{tmj}^{Q}+C_{tmj}}100\%$$ (31)

## 3. Results

### 3.1 Land use and land cover mapping

**Fig 2:**
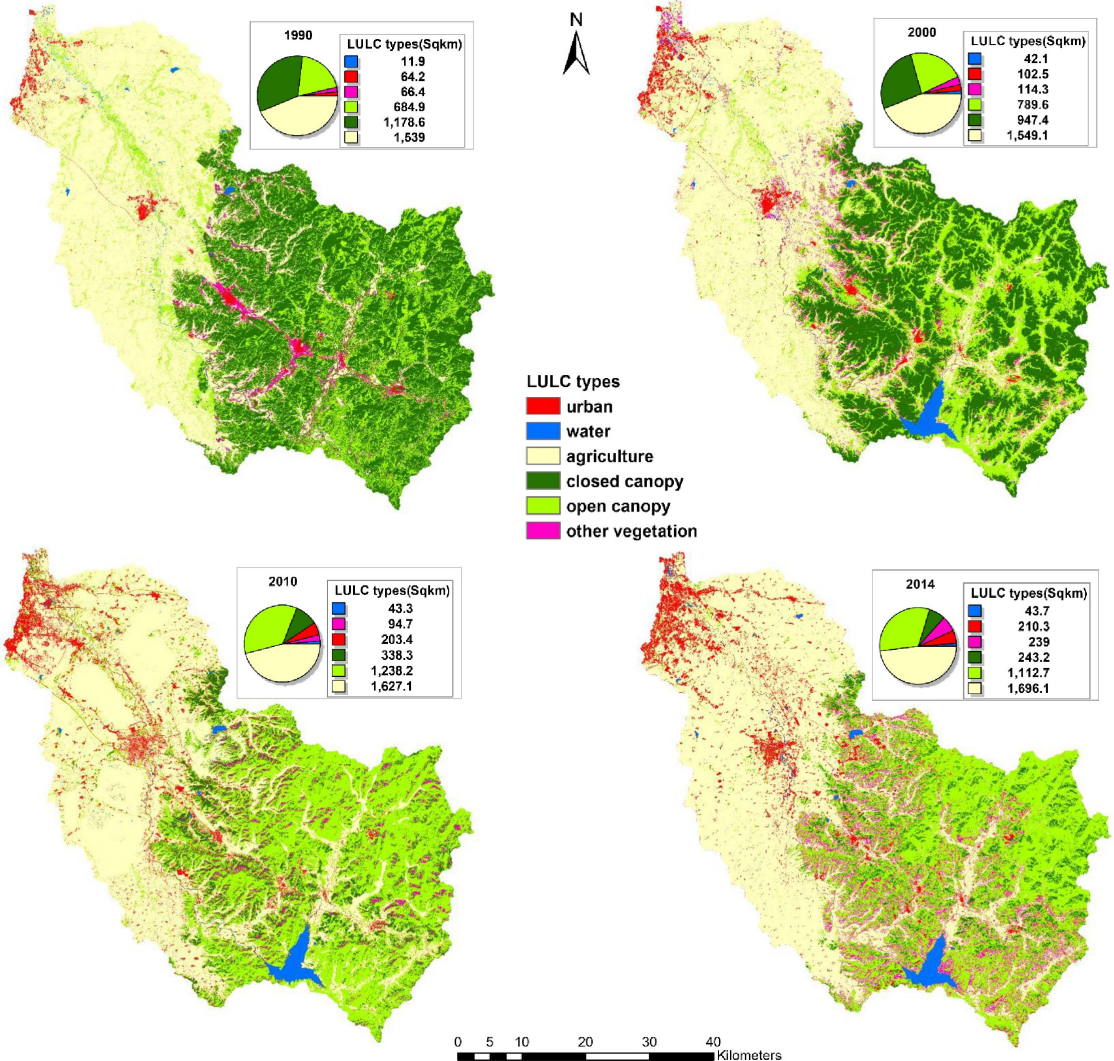
LULC maps of the watershed at four time points.

Four LULC maps for URB, WAT, AGR, CLC, OPC and OTV of the years 1990, 2000, 2010 and 2014 were produced to investigate the LULCC at four time points (Fig 2).

### 3.2 Accuracy assessment

The overall accuracies of all the four maps examined with ground-truth points for each time point were greater 89% and generated kappa statistics of more than 84% suggesting accuracy between the classifications made and the ground reference information. Table 3 displays the individual accuracy of the LULC classes quantified using the producer’s accuracy (omission error) and user’s accuracy (commission error) which was based on confusion matrix. The minimum of about 67% record for OTV was accepted because of its similarity with OPC and CLC classes and AGR.

**Table 3:** Classification accuracy values for LULC maps (%).

LULC Classes	1990		2000		2010		2014	
	PA^a^	UA^b^	PA	UA	PA	UA	PA	UA
URB	100	90	86.67	86.67	100	86.67	92.31	80
WAT	100	93.33	100	93.33	93.75	100	92.31	80
AGR	85.71	80	91.67	73.33	92.86	86.67	75	80
CLC	100	90	83.33	100	81.82	90	88.89	80
OPC	87.5	70	81.82	90	83.33	100	69.23	80
OTV	66.67	80	72.73	80	77.78	70	72.73	80
Overall Accuracy (%)	84.00		86.67		89.33		81.33	
Kappa Coefficient	0.8062		0.8394		0.8712		0.7749	

^**a**^Producer Accuracy

^**b**^ User Accuracy.

### 3.3 Intensity analysis

#### 3.3.1 Time interval

**Fig 3:**
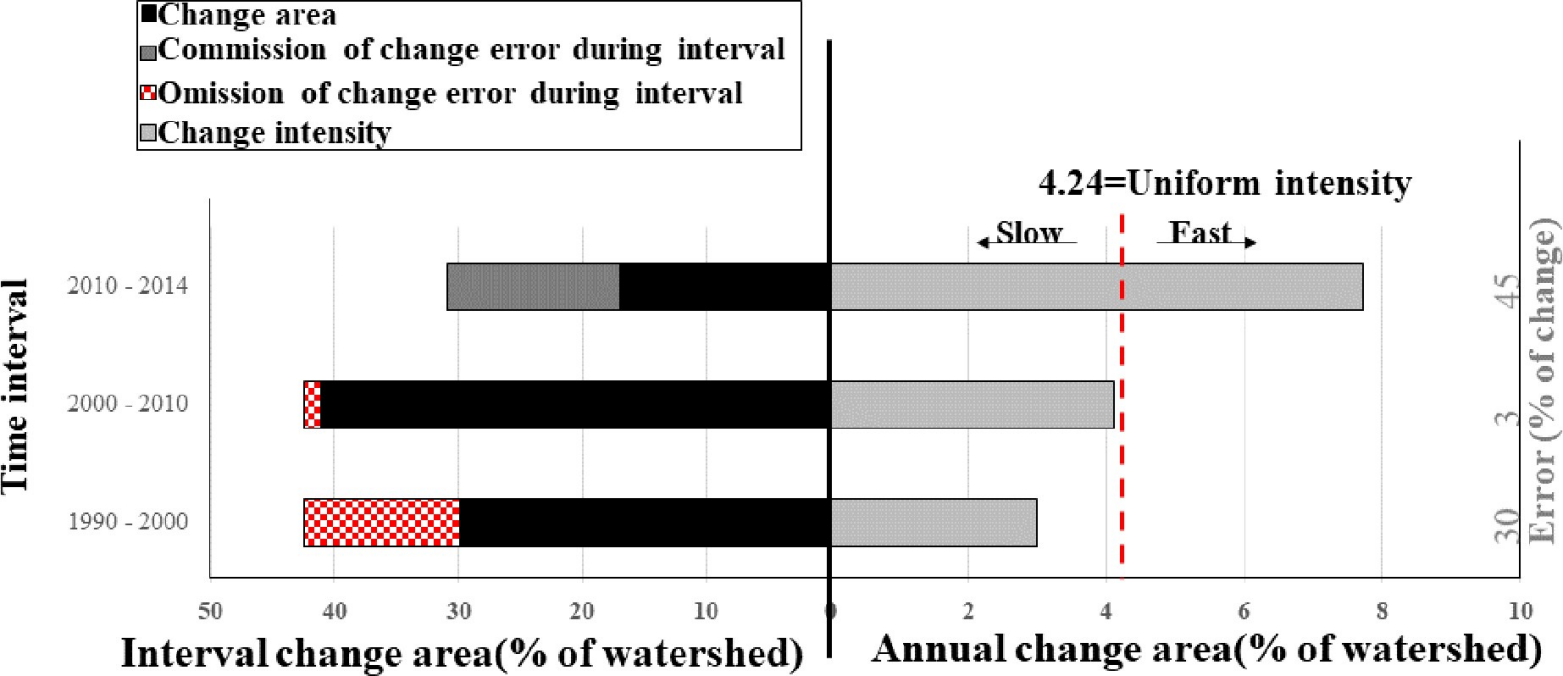
Interval change area along with hypothetical errors that could give reasons for the deviations from UI and yearly change area of the three-time intervals: 1990–2000, 2000–2010 and 2010–2014.

The yearly change is faster from 2010–2014 with a change percentage of 31% in the watershed, though the interval change was the smallest (Fig 3). The yearly change during the intervals 1990–2000 and 2000–2010 are slow with 30% and 41% change in the watershed area respectively. Given that the yearly changes for all intervals are constant during the time extent, the yearly rate of LULCC would be equal to 4.24%, as indicated by the red dashed line in Fig 3. However, the rate of LULCC in the watershed revealed non-uniformity. The right side of the UI line in Fig 3 shows, a faster speed of yearly change, 7.72% of the interval’s watershed, during 2010–2014. The left side of the UI line displayed a slow rate of annual change, 2.99% and 4.10% of the interval’s watershed, during 1990–2000 and 2000–2010 respectively.

#### 3.3.2 Category level

**Fig 4:**
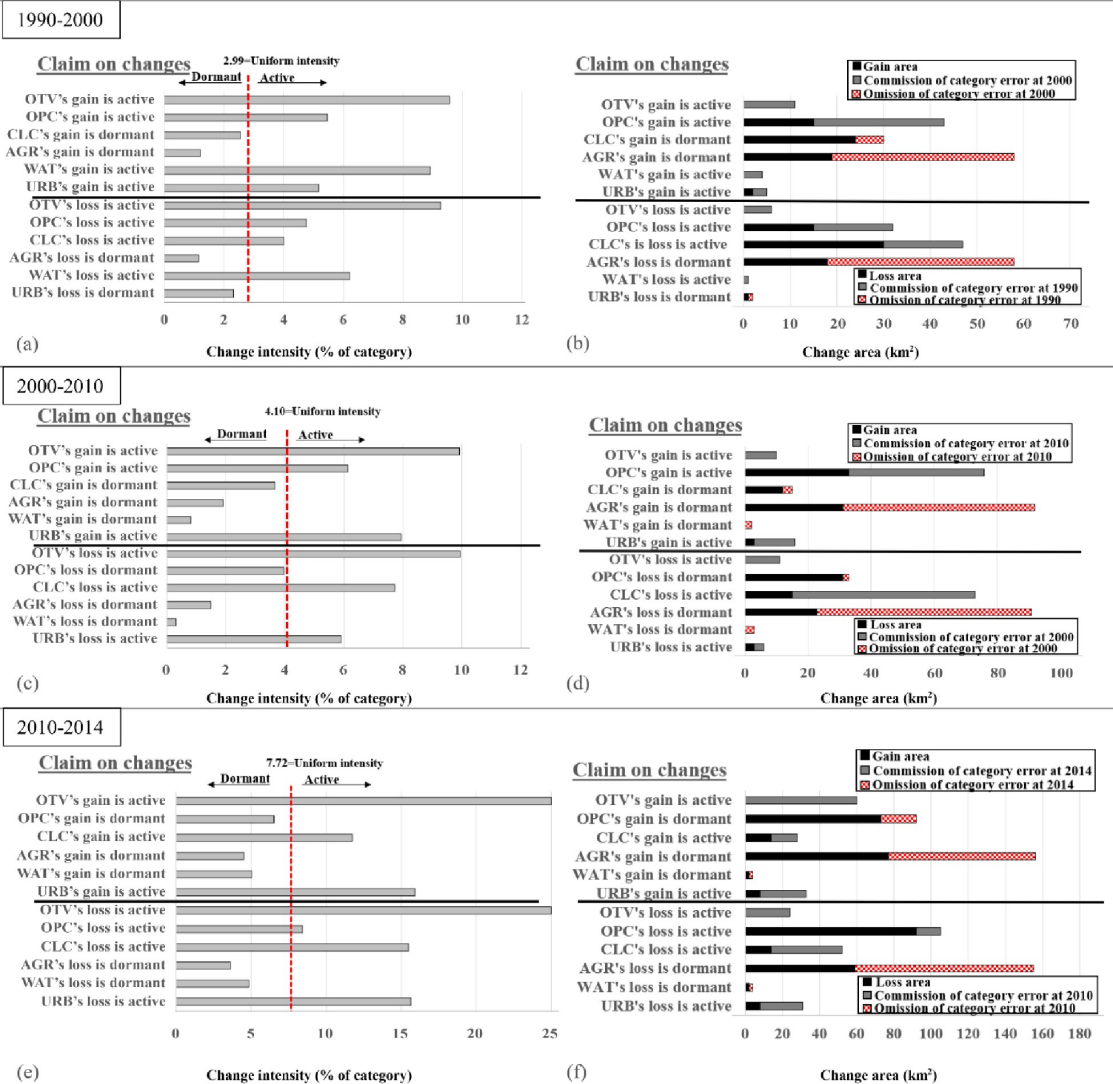
Category-stage gain and loss intensities given the detected alteration through three-time intervals. Gain intensity is a proportion of the class at the final time point, while the loss intensity is a proportion of the class at the initial time point.

The change intensity on gains and losses are shown in Fig 4A, 4C and 4E while Fig 4B, 4D and 4F indicates the area of gains and losses along with errors that could explain the differences between the change areas and the uniform change intensities at the category level. If a class’s bar goes beyond the UI line, then the gain or loss intensity for that class is active, but if a bar stops before the UI line, then the intensity is dormant. Six active categorical changes in terms of loss and gain during 1990–2000: URB gain, WAT gain, WAT loss, CLC loss, OPC gain, OPC loss, OTV gain and OTV loss are shown in Fig 4. All other gains and losses show dormancy. For 2000–2010, six active categorical changes are revealed (Fig 4C): URB gain, URB loss, CLC loss, OPC gain, OTV gain and OTV loss. All other gains and losses exhibit dormancy. Similarly, seven active categorical changes were discovered during the 2010–2014 interval: URB gain, URB loss, CLC gain, CLC, loss, OPC loss, OTV gain and OTV loss. The extra gains and losses revealed dormancy during this interval (Fig 4E). The observed change area for each active class is the sum of uniform change and commission error at each interval, while the observed change area for each dormant class is the sum of the observed change and omission error at each interval (Fig 4B, 4D and 4F). AGR, OPC and CLC have the largest gains and losses, while URB and OTV have the highest intensities with small sizes of losses and gains, which account for their high intensities. AGR loss and gain intensities are dormant because of the large extent of AGR. CLC and OPC loss intensities are active for all the intervals, while the gain in CLC is dormant for the first two intervals and active at the last interval. This dormancy of CLC gain accounts for OPC active gain in the first two intervals and dormant in the last interval (Fig 4).

#### 3.3.3 Transition analysis

**Fig 5:**
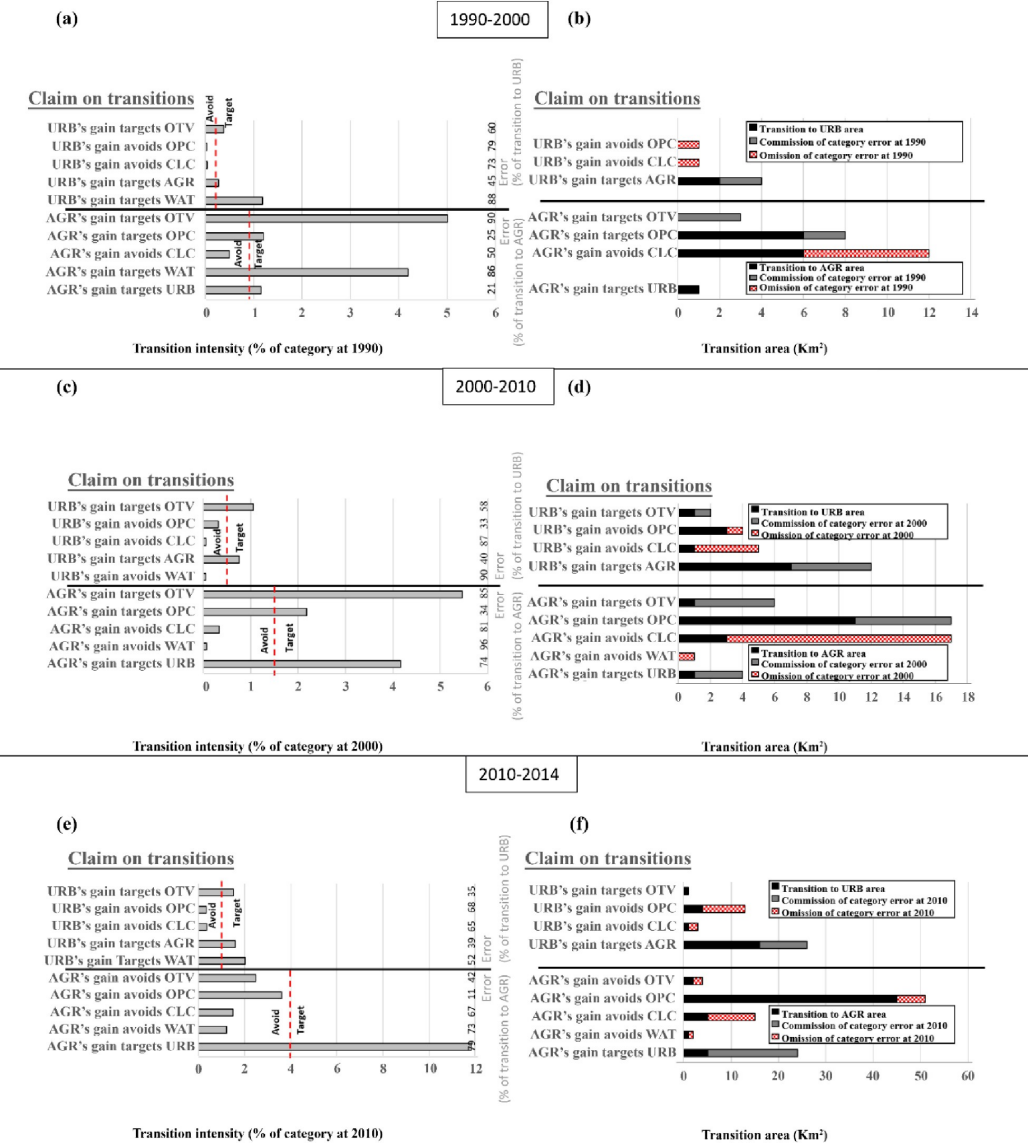
Transition level analysis for URB gain and AGR gain during the three time intervals. Graphs on right show transition intensity and graphs on left show sizes of transitions along with hypothetical errors at the initial time point map that could account for deviations from uniform transition intensities indicated by the dashed uniform line.

Considering the rapid expansion nature of urban areas and agriculture activities after China’s 1978 economic reforms in the Ashi watershed, this research focused on the transition from CLC, OPC, OTV and WAT to URB and AGR. The transition from CLC, OPC, OTV and WAT to URB and AGR are shown in Fig 5. The six graphs (Fig 5) illustrate the analysis of gain to both URB and AGR for the three-time intervals. Each interval has two graphs showing the transition intensity and size of transitions together with hypothetical errors at the early time point map that could explain the difference between transition area and hypothesized transition intensities indicated by the dashed UI line. The gain of a class targets a losing class if the bar of that class extends beyond the UI line, whereas a class gain avoids a losing class if the bar ends before the UI line. Gains in URB targets OTV, AGR and avoids OPC and CLC in all the time intervals (Fig 5A, 5C and 5E). Thus, the transition from OPC, CLC, OTV and AGR to URB in terms of target or avoid is stationary. This means the pattern of change is the same for all the time intervals given the gain of URB. Also, AGR gains targets URB during all the intervals, but targets OTV and OPC for the first and second intervals. AGR gains nearly targeted OPC in the third interval because the intensity in OPC transition is slightly less than the UI while avoiding CLC for all the intervals. Hence, the change from URB and CLC to AGR is stationary for all intervals while the change from OTV and OPC to AGR is stationary for the first and second intervals given the gain of AGR. Uniform transition plus errors in the initial time points of the intervals 1990–2000, 2000–2010 and 2010–2014 respectively that could account for deviations from uniform transition intensities are indicted in Fig 5B, 5D and 5F. The observed gains of URB and AGR as target is the sum of a uniform yearly transition area and commission of the losing classes errors at the initial time point; while observed gains of URB and AGR as avoid, is the sum of uniform yearly transition areas and omission of the losing classes errors at the initial time point in each interval. These errors are shown under the error analysis section of the results.

**Fig 6:**
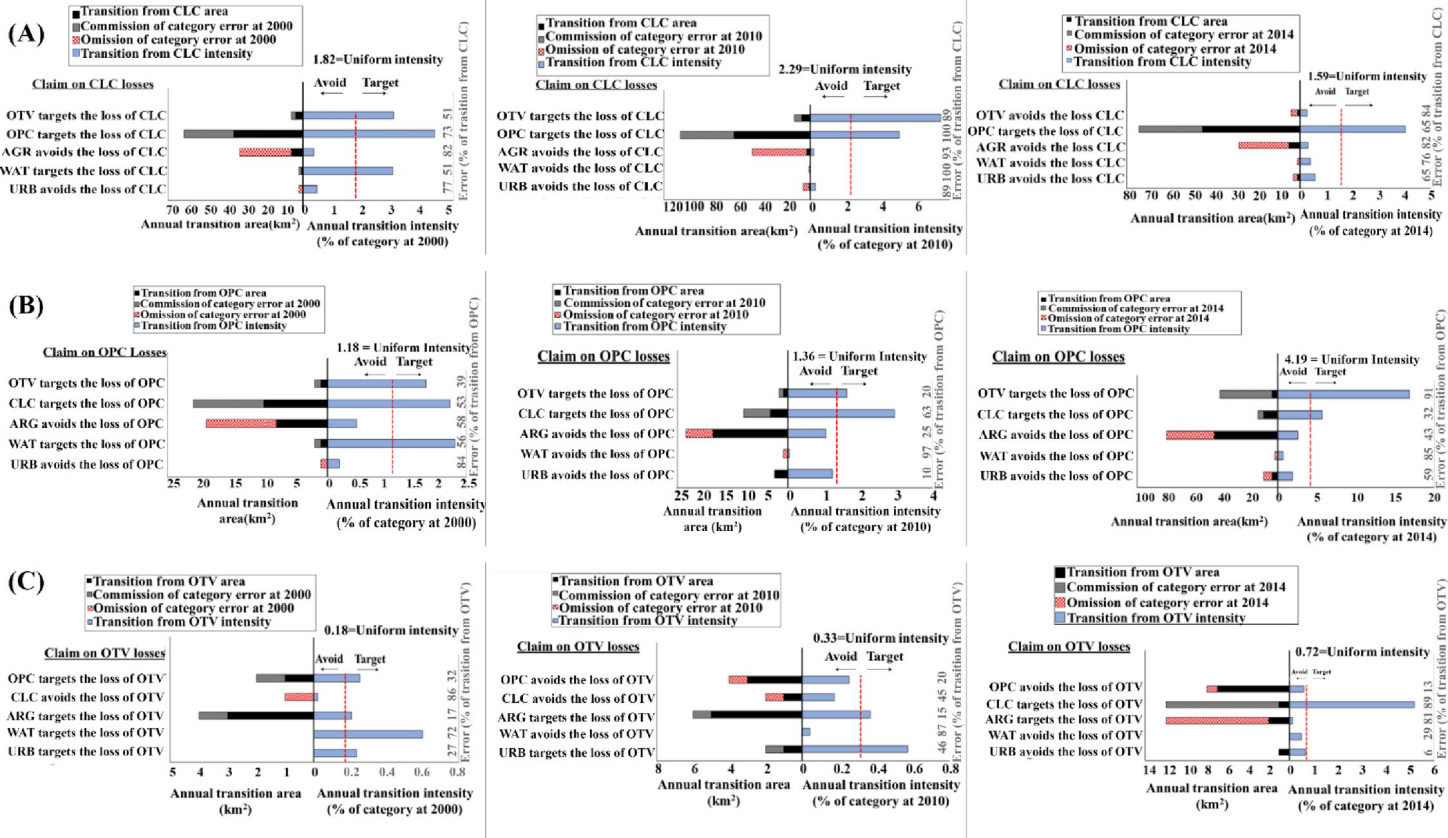
Intensity analysis for transition from CLC, OPC and OTV, given CLC, OPC and OTV observed losses during 1990–2000, 2000–2010 and 2010–2014.

URB and AGR avoids loss in CLC and OPC for all time intervals whilst OTV targets the losses of OPC and CLC, except the losses of CLC for 2010–2014 (Fig 6A and 6B). URB targets the losses of OTV for all intervals, while AGR targets the losses of OTV for the first and second time interval (Fig 6). The transition from CLC, OPC and OTV to URB is stationary given the loss of CLC, OPC and OTV. The transition from CLC, OPC and OTV to AGR is stationary for the first and second time intervals given the losses of CLC, OPC and OTV.

### 3.4 Error analysis

The error analysis indicates that commission of change error in 13.91% of the watershed could explain the change during 2010–2014 occurring faster than the uniform rate of change. The intensity of commission of change error detected for 2010–2014 was 45%. Also, omission of change error in 12.53% and 1.38% of the watershed during 1990–2000 and 2000–2010 respectively could account for their change appearing slower than the uniform rate of change. The intensity of omission of change error of the detected change for 1990–2000 and 2000–2010 was 30% and 3% respectively (Fig 3)

For errors at the category level, the top of Fig 4B displays claim on gain, where class errors in 13% of the year 2000 map could clarify all differences between the detected gain intensities and the UI during 1990–2000. The bottom of Fig 4B shows claim on losses where class errors in 12% of the 1990 map might explain all differences between the detected loss intensities and the UI during 1990–2000. For 2000–2010, the top of Fig 4D indicates claim on gain where class errors in 19% of the 2010 map might account for all differences between the detected gain intensities and the uniform intensity. Claim on losses is exhibited by the bottom of Fig 4D, where class errors in 21% of the 2000 map could explain all differences between the detected loss intensities and the uniform intensity during 2000–2010.

**Fig 7:**
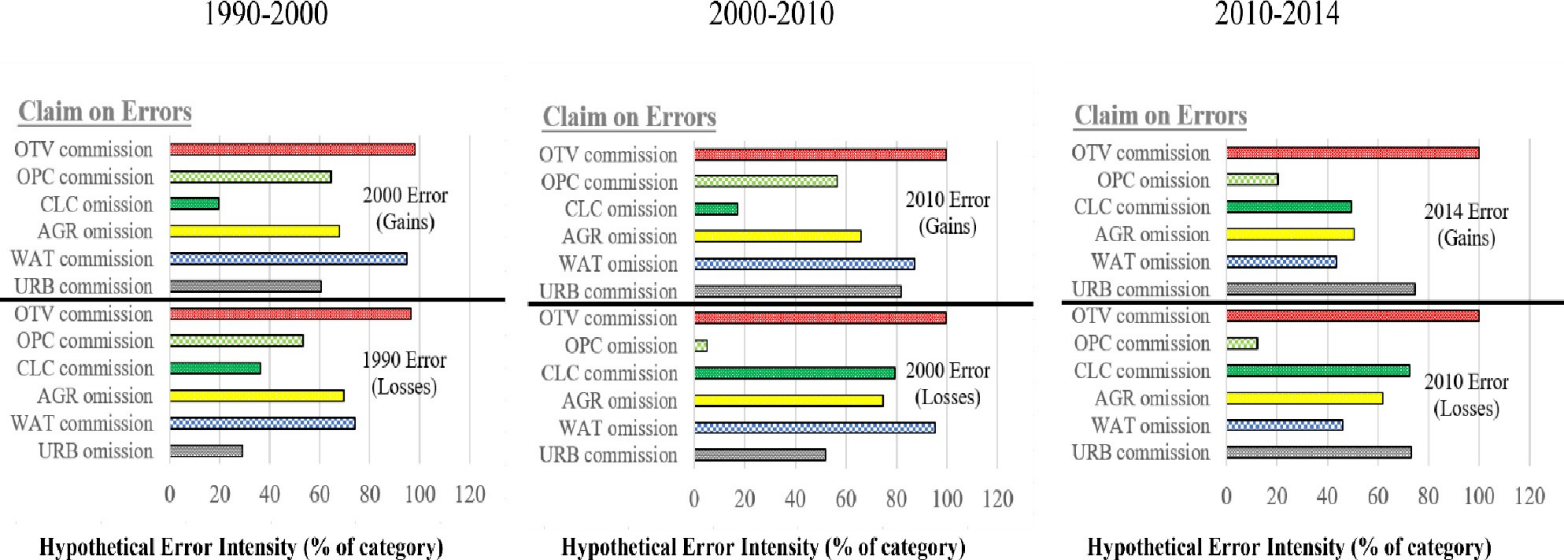
Intensities of errors in the change area that may account for deviations from uniform class gains and losses. In the event that the real intensities of errors are not greater than the values in indicated, it evidences the non-uniformity of the gains and losses at the category level.

Similarly, the top of Fig 4F denotes gain claims where class errors in 11.2% of the 2014 map could explain all differences between the detected gain intensities and the uniform intensity during 2010–2014. The bottom of Fig 4F demonstrates losses where class errors in 11% of the 2010 map could account for differences between the detected loss intensities and the uniform intensity during 2010–2014. Fig 7 shows the error intensities that could explain for the differences in Fig 4. The bars for the final time point at the upper part of the graphs for each interval indicates the intensities of the errors that could account for divergence from a uniform gain, while the bars for the initial time point at the lower part of the graphs at each interval shows the intensities of the mistakes that could explain the divergence from a uniform loss. Precisely, if the real commission error intensity for URB in 2000 < 60% then it indicates that URB is actively gaining. Whereas, if the real omission error intensity for URB in 1990 < 29%, then it indicates that URB is dormant regarding losses during 1990–2000. The same logic covers all categories at each interval.

The hypothetical error in the 1990 map that explains the divergences from uniform transition intensities (UTI) is depicted in Fig 5B. Transition errors in 0.6% and 1.6% of the 1990 map could explain the divergences from uniform transitions given URB gain and AGR gain respectively during 1990–2000. The hypothetical error in the 2000 map that could account for the divergences from UTI is illustrated in Fig 5D. Transition errors in 1.5% and 4% of the 2000 map could explain the divergences from uniform transitions given gain in URB and AGR respectively during 2000–2010. Similarly, the hypothetical error in the 2010 map that could explain the divergence from UTI is depicted in Fig 5F. Transition errors in 1.2% and 2.1% of the 2010 map could account for divergences from uniform transitions given gain in URB and AGR respectively during 2010–2014.

## 4. Discussion

### 4.1 Patterns and processes in LULCC from IA

The time interval analysis shows that if LULC changes across the watershed were distributed uniformly throughout all the intervals, then the rate of LULCC would amount to 4.24%. However, the rate of change was not uniform because it has been accelerating in subsequent intervals with the fastest rate taking place during 2010–2014 followed by 2000–2010 and then 1990-2000.This speed of the LULCC is consistent with the emergence of China’s market-oriented reforms, which stimulated the development of modern industries especially food and agriculture product processing industries [38, 39] in the watershed. This development propels LULCC as human and economic actions upsurge. Similar observations were made in southeast China during the 1986–1996, 1996–2002, 2002–2007 and 2007–2010 intervals, where LULCC accelerated with successive intervals [25].

During all the time intervals, the URB actively gained with obvious active losses in CLC and OPC because of the high demand for land to construct urban facilities in the watershed. This is evidenced by accelerated urban expansion rate in China; 17.9% in 1978, 26.4% in 1990, 36.2% in 2000, 49.9% in 2010 and 56.1% in 2015, [40–42] over the past 30 years of economic reform. Moreover, a large resettlement programme in the Heilongjiang Reclamation Area (HRA) where the study area is located, opened up land for cultivation purposes and development in urban zones [38]. Also URB, had dormant loss in the first interval and active loss in the second and third intervals which indicates efforts by both national and regional authorities to promote land use efficiency, by reducing urban construction planning by 74% [43] during these periods. AGR gains and losses indicated dormancy during all the time intervals, which demonstrates the large dormant category phenomena [23] and accounts for majority of the area at four-time points. The active losses of CLC and OPC during the intervals are mainly due to the clearing of forest for construction and agricultural activities in the watershed which is currently noted as a major agricultural and rapid urbanization zone. The active loss of OPC might be attributed to the active loss of CLC, since CLC have to be disturbed for human activities, which results in OPC formation. This finding is similar to studies by [44] where a modification from CLC to OPC by human activities was noticed. Moreover, CLC is dormant while OPC is active in the first and second intervals implying that as CLC is losing in the first and second intervals, it paves way for OPC gains during these intervals.

The systematic targeting of transition from AGR, OTV and WAT to URB during all the time intervals in the watershed is linked to the fact that China’s rapid urban sprawl has prohibited expansion of agricultural lands [45].This can also be partly due to the existence of agricultural activities, grassland and water bodies in flat areas, where towns are likely to be located [46–49]. Therefore, as URB areas extend, it is possible to gain from OTV, AGR and WAT. This explains why most gain in URB land adjoining cities such as Acheng, Chenggaozizhen, Xingfuzhen and Dongfanghongcu come from agriculture and other vegetation. This observation is in line with [25]. The avoiding transition from CLC and OPC to URB is in line with the Chinese environmental policy. In the 1990s the ecological functions of forest, and other natural land covers were recognized nationwide and therefore the ‘grain to green policy’ were adopted, which lessened the rate of reclamation of natural land cover [39]. Similarly, the gain in AGR targeted OTV, OPC, and URB during the first and second intervals, but only targeted URB during the third interval. This is associated with the ongoing effort by the Chinese government to retain lands for agriculture activities, since the country’s cultivation areas have reduced due to their withdrawal from agriculture for building manufacturing facilities. In addition, converting rural land into urban areas put stress on ecological systems since numerous ecosystem services provided by agricultural land disappear in the process of conversion [50].

### 4.2 Error analysis

The OTV commission hypothetical error during the three intervals is noticeably bigger than the other errors. There is therefore strong evidence for the deduction of active gains and losses in OTV than the other deductions on gains and losses indicated in Fig 7. During 1990–2000, CLC omission error is less than the other errors and thus, there is weak evidence for the deduction that CLC is dormant with respect to gains than the other deductions regarding gains. Similarly, the URB omission hypothetical error illustrates weak evidence when compared to the other hypothetical errors for URB being dormant concerning losses. The commission hypothetical error of CLC is more than the other hypothetical errors during 2000–2010, which concludes that there is strong evidence for active CLC losses than the other deductions on losses. OPC omission and URB commission hypothetical errors are less than the other hypothetical errors thus; there is weak evidence for the deduction that with respect to losses, OPC and URB are dormant and active respectively when compared to the other deductions regarding categorical losses during 2000–2010.

The transition stage of the IA reveals that URB gain from OTV and AGR is stationary because the deviation from the uniform transition is positive during all the three time intervals. However, when the strength of the evidence is quantified, the error analysis shows that the evidence is weak because only 2km^2^ of commission of AGR error and 0km^2^ of commission of OTV error in 1990 could account for the positive deviation during 1990–2000. Additionally, only 5km^2^ of commission of AGR error and 1km^2^ of commission of OTV error in 2000 could account for the positive deviation during 2000–2010. Finally, only 16km^2^ of commission of AGR error and 0km^2^ of commission of OTV error at 2010 could account for the positive deviation during 2010–2014. Considering that the total watershed area is about 3545km^2^, it is rational to conclude that URB gain is non-stationary with respect to OTV and AGR during all the three time intervals [22].

Likewise, the transition from URB and CLC to AGR is stationary. Nonetheless the error analysis suggest that the evidence is weak because 0km^2^ of commission of URB error in 1990, 3km^2^ of commission of URB error in 2000; and 1km^2^ of commission of URB error in 2010 could account for the positive deviations during all the three intervals. Besides, only 6km^2^ of omission of CLC error in 1990, 14km^2^ of omission of CLC error in 2000 and 10km^2^ of omission of CLC error in 2010 could account for the negative deviation during all the three intervals compared to the total area of the watershed.

The conclusion drawn from the IA of the sampled data, that the transition from OTV and OPC to AGR during the first and second interval is stationary strengthens analyzing the error to highlight weak evidence. This is because only 3km^2^ of commission of OTV error and 2km^2^ of commission of OPC error in 1990, as well as only 5km^2^ of commission of OTV error and 6km^2^ commission of OPC error in 2010 could account for the positive deviations during the first and second intervals respectively. Considering the size of the study area, the hypothetical transition errors are smaller than the suspected errors in the data.

## 5. Conclusion

The application of IA to data from the Ashi watershed reveals seeming change intensities in six LULC classes comprising URB, WAT, AGR, CLC, OPC and OTV. The change intensities are not uniform, assuming changes were to be distributed uniformly across the entire temporal and watershed extent. The overall LULCC in the watershed has been accelerating, reflecting rapid economic development in successive intervals. AGR, OPC and CLC have the largest gains and losses, while URB and OTV have highest intensities but relatively smaller losses and gains. AGR is dormant in both losses and gains due to it covering a large area. URB gains mainly from OTV, AGR, and avoids OPC and CLC. Transition from URB and CLC to AGR is stationary for all time intervals while the transition from OTV and OPC to AGR is stationary for the first and second intervals given the gain in AGR.

The minimum hypothetical errors that could explain the non-uniformity of the change intensities were measured. These errors revealed the strength of the evidence for the deviations from uniform intensities based on the sizes of errors. This study therefore concludes that OTV is active in terms of gains and losses during all the intervals but has weak evidence; CLC gain and URB loss are dormant during 1990–2000 but has weak evidence; CLC loss is active during 2000–2010 and has strong evidence whilst OPC and URB losses are dormant during 2000–2010 but has weak evidence. Additionally, the transition from OTV and AGR to URB is stationary for all intervals but has weak evidence whilst the transition from URB and CLC to AGR is stationary for all intervals but has weak evidence.

Strong evidence for a specific deviation from the applicable hypothesized UI is indicated by large hypothetical error. The evaluation of the consequences of hypothetical errors in this study indicates the strength of evidence for each of the results from IA. It is recommended that users of the IA concept consider assessing their map errors, since there is limited ground information on past time point data. These errors will indicate the strength of evidence for deviations and reveal patterns that increase insight into LULCC processes.

## Supporting information

S1 Table Area of LULC classes of the classification (km^2^). (DOCX)

S2 Table Transition matrix of LULC types from 1990 to 200(km^2^). (DOCX)

S3 Table Transition matrix of LULC types from 2000 to 2010(km^2^). (DOCX)

S4 Table Transition matrix of LULC types from 2010 to 2014(km^2^). (DOCX)

S1 File Spreadsheet for intensity analysis. (XLSM)

S2 File Descriptions of supplementary material files. (DOCX)
