# Supplementary material for: Evidence and impact of map error on land use and land cover dynamics in Ashi River watershed using intensity analysis
Source: PLoS One. 2020 Feb 20;15(2):e0229298. doi: 10.1371/journal.pone.0229298 (PMC7032735; doi:10.1371/journal.pone.0229298)
Supplement: S1 Table — (DOCX) [file pone.0229298.s001.docx]

**Table 1** Area of LULC classes of the classification (Km^2^)

| Area in Given year | URB | WAT | AGR | CLC | OPC | OTV |
| --- | --- | --- | --- | --- | --- | --- |
| 1990 | 64.2 | 11.9 | 1539 | 1178.6 | 684.9 | 66.4 |
| 2000 | 102.5 | 42.1 | 1549.1 | 947.4 | 789.6 | 114.3 |
| 2010 | 203.4 | 43.3 | 1627.1 | 338.3 | 1238.2 | 94.7 |
| 2014 | 210.3 | 43.7 | 1696.1 | 243.2 | 1112.7 | 239 |
|  |  |  |  |  |  |  |
