# Supplementary material for: Evidence and impact of map error on land use and land cover dynamics in Ashi River watershed using intensity analysis
Source: PLoS One. 2020 Feb 20;15(2):e0229298. doi: 10.1371/journal.pone.0229298 (PMC7032735; doi:10.1371/journal.pone.0229298)
Supplement: S2 Table — (DOCX) [file pone.0229298.s002.docx]

**Table 2.**

Transition Matrix of LULC types from 1990 to 2000 (Km^2^)

| LULC Classes | | 2000 Final state | | | | | | | |
| --- | --- | --- | --- | --- | --- | --- | --- | --- | --- |
|  |  | URB | WAT | AGR | CLC | OPC | OTV | Total | Loss |
| 1990 Initial state | URB | 49.3 | 0 | 7.35 | 0 | 7.55 | 0 | 64.2 | 14.9 |
|  | WAT | 1.4 | 4.5 | 5 | 0.4 | 0.3 | 0.3 | 11.9 | 7.4 |
|  | AGR | 42.2 | 12.1 | 1362.5 | 27.2 | 42.2 | 52.8 | 1539 | 176.5 |
|  | CLC | 4.9 | 13.1 | 58.6 | 705.8 | 360.3 | 35.9 | 1178.6 | 472.8 |
|  | OPC | 2.2 | 9.8 | 82.35 | 211.6 | 358.45 | 20.5 | 684.9 | 326.45 |
|  | OTV | 2.5 | 2.6 | 33.3 | 2.4 | 20.8 | 4.8 | 66.4 | 61.6 |
|  | Total | 102.5 | 42.1 | 1549.1 | 947.4 | 789.6 | 114.3 | 3545 |  |
|  | Gain | 53.2 | 37.6 | 186.6 | 241.6 | 431.15 | 109.5 |  | 1059.65 |
