# Supplementary material for: Evidence and impact of map error on land use and land cover dynamics in Ashi River watershed using intensity analysis
Source: PLoS One. 2020 Feb 20;15(2):e0229298. doi: 10.1371/journal.pone.0229298 (PMC7032735; doi:10.1371/journal.pone.0229298)
Supplement: S3 Table — (DOCX) [file pone.0229298.s003.docx]

**Table 3.**

Transition Matrix of LULC types from 2000 to 2010(Km^2^)

| LULC Classes | | 2010 Final state | | | | | | | |
| --- | --- | --- | --- | --- | --- | --- | --- | --- | --- |
|  |  | URB | WAT | AGR | CLC | OPC | OTV | Total | Loss |
| 2000 Initial state | URB | 41.9 | 2.3 | 42.7 | 3.1 | 5.6 | 6.9 | 102.5 | 60.6 |
|  | WAT | 0.2 | 40.8 | 0.3 | 0.1 | 0.7 | 0 | 42.1 | 1.3 |
|  | AGR | 117.7 | 0.9 | 1316.9 | 14 | 97.6 | 2.0 | 1549.1 | 232.2 |
|  | CLC | 6.2 | 0 | 32.4 | 214.6 | 621.9 | 72.3 | 947.4 | 732.8 |
|  | OPC | 25.3 | 0.2 | 172.4 | 100.3 | 475.3 | 16.1 | 789.6 | 314.3 |
|  | OTV | 12.1 | 0.2 | 62.4 | 6.2 | 32.7 | 0.7 | 114.3 | 113.6 |
|  | Total | 203.4 | 43.3 | 1627.1 | 338.3 | 1238.2 | 94.7 | 3545 |  |
|  | Gain | 161.5 | 2.5 | 310.2 | 123.7 | 762.9 | 94 |  | 1454.8 |
