# Supplementary material for: Evidence and impact of map error on land use and land cover dynamics in Ashi River watershed using intensity analysis
Source: PLoS One. 2020 Feb 20;15(2):e0229298. doi: 10.1371/journal.pone.0229298 (PMC7032735; doi:10.1371/journal.pone.0229298)
Supplement: S4 Table — (DOCX) [file pone.0229298.s004.docx]

**Table 4.**

Transition Matrix of LULC types from 2010 to 2014(Km^2^)

| LULC Classes | | 2014 Final state | | | | | | | |
| --- | --- | --- | --- | --- | --- | --- | --- | --- | --- |
|  |  | URB | WAT | AGR | CLC | OPC | OTV | Total | Loss |
| 2010 Initial state | URB | 76.1 | 3.8 | 96.3 | 3.7 | 18.9 | 4.6 | 203.4 | 127.3 |
|  | WAT | 3.5 | 34.9 | 2.1 | 0.4 | 1.5 | 0.9 | 43.3 | 8.4 |
|  | AGR | 103.7 | 2.1 | 1389.7 | 2.9 | 62.5 | 66.2 | 1627.1 | 237.4 |
|  | CLC | 4.9 | 0.7 | 20.2 | 128.8 | 181.1 | 2.6 | 338.3 | 209.5 |
|  | OPC | 16.4 | 1.3 | 178.4 | 56.5 | 820.9 | 164.7 | 1238.2 | 417.3 |
|  | OTV | 5.7 | 0.9 | 9.4 | 50.9 | 27.8 | 0 | 94.7 | 94.7 |
|  | Total | 210.3 | 43.7 | 1696.1 | 243.2 | 1112.7 | 239 | 3545 |  |
|  | Gain | 134.2 | 8.8 | 306.4 | 114.4 | 291.8 | 239 |  | 1094.6 |
