# Supplementary material for: Evidence and impact of map error on land use and land cover dynamics in Ashi River watershed using intensity analysis
Source: PLoS One. 2020 Feb 20;15(2):e0229298. doi: 10.1371/journal.pone.0229298 (PMC7032735; doi:10.1371/journal.pone.0229298)
Supplement: S2 File — (DOCX) [file pone.0229298.s006.docx]

**Description of each supplementary material files**

**1.Transition Matrices file**: This file contains data in cross-tabulation format, that represents the change in Land use and land cover of the watershed, which are computed from the classified LULC maps for three-time intervals, 1990-2000, 2000-2010 and 2010-2014 and serve as an input data for the algorithms of the intensity analysis. Tables 1, 2 and 3 contain the transition matrix for each of the intervals; 1990-2000, 2000-2010 and 2010-2014 respectively. Each matrix has a total column at the right that gives the coverage area of each LULC type at the initial time point, and a total row at the bottom that gives the coverage area of each LULC type at the final time point of each interval. Also, the matrix for each time interval shows the flow of each LULC type by presenting a column of gross losses at the far right and a row of gross gains at the very bottom.

**2.Intensity Analysis Spreadsheet file**: This file contains the intensity analysis computer program with our dataset and the results in output of intensity analysis algorithms. We feed in our transition matrices values in the InputMatrix sheet of the computer program of intensity analysis and run it to obtain the values of the equations used in our study. This computer program can be obtained from https://sites.google.com/site/intensityanalysis/free-computer-programs.
